# A Comparative Study of Intramolecular Mobility of Single Siloxane and Carbosilane Dendrimers via Molecular Dynamics Simulations

**DOI:** 10.3390/polym10080838

**Published:** 2018-07-30

**Authors:** Andrey O. Kurbatov, Nikolay K. Balabaev, Mikhail A. Mazo, Elena Yu. Kramarenko

**Affiliations:** 1 Faculty of Physics, Lomonosov Moscow State University, Moscow 119991, Russia; kurbatov@polly.phys.msu.ru; 2 A. N. Nesmeyanov Institute of Organoelement Compounds RAS, Moscow 119991, Russia; 3 Institute of Mathematical Problems of Biology, Keldysh Institute of Applied Mathematics RAS, Pushchino, Moscow 142290, Russia; balabaevnk@gmail.com; 4 Semenov Institute of Chemical Physics RAS, Moscow 119991, Russia; mikhail.mazo1@gmail.com

**Keywords:** dendrimers, siloxane dendrimers, carbosilane dendrimers, molecular dynamics simulations, intramolecular dynamics

## Abstract

A comparative analysis of intramolecular dynamics of four types of isolated dendrimers from the fourth to the seventh generations belonging to the siloxane and carbosilane families, differing in spacer length, core functionality, and the type of chemical bonds, has been performed via atomic molecular dynamics simulations. The average radial and angular positions of all Si branching atoms of various topological layers within the dendrimer interior, as well as their variations, have been calculated, and the distributions of the relaxation times of their radial and angular motions have been found. It has been shown that the dendrons of all the dendrimers elongate from the center and decrease in a solid angle with an increasing generation number. The characteristic relaxation times of both angular and radial motions of Si atoms are of the order of a few nanoseconds, and they increase with an increasing generation number and decrease with temperature, with the angular relaxation times being larger than the radial ones. The relaxation times in the carbosilanes are larger than those in the siloxanes. The rotational angle dynamics of the carbosilane dendrimers show that the chain bending is mainly realized via trans-gauche transitions in the Si branching bonds.

## 1. Introduction

Dendrimers are hyperbranched molecules with a regular structure [1,2,3]. Their behavior is unique due to the specific organization of their molecular structure, high monodispersity, and high functionality. Recent progress in chemical synthesis has allowed one to reach a large variety of dendrimer compositions [4,5,6,7,8,9,10,11,12,13] available for various envisioned applications in different areas of physics [6,7], chemistry [8,9], biology, and medicine [10,11,12,13].

Along with single dendrimers in dilute solutions [14,15,16,17,18,19,20,21,22,23], much attention is attracted nowadays to concentrated solutions and melts [24,25,26,27,28,29,30,31,32,33,34], where intermolecular interactions of dendrimer molecules are significant. It is expected that the tree-like molecular morphology of dendrimers would distinguish their behavior from that of conventional linear polymers. Indeed, a number of novel unusual phenomena has recently been observed in dendrimer melts that do not have any strict fundamental description. One such phenomenon is an unprecedented jump in the viscosity of high-generation carbosilane dendrimer melts [31]. It has been found that while low-generation dendrimer melts are Newtonian liquids, high-generation dendrimers demonstrate solid-like behavior. The transition between these states is very sharp; it is realized within one generation step (from the fifth to the sixth generation), and it is accompanied by a six-orders-of-magnitude jump of the melt viscosity [31]. The dendrimer specificity also manifests itself in NMR spectra of melts of polybutylcarbosilane dendrimers from the fourth to the sixth generation [32]; in particular, the spectrum of the generation-6 (G6) dendrimers is characterized by a single unresolved broad line in the whole temperature range above the glass transition temperature, which suggests the presence of an anomalous phase state of G6 dendrimers with restricted molecular mobility. A similar anomalous phase state has been detected in generation-5 (G5) dendrimers at enhanced temperatures above 473 K [32]. The physical reason for this behavior is still unclear, and further studies are needed.

In this respect, computer simulations are a powerful tool to elucidate the impact of the dendrimer structure on their unusual properties. In particular, computer simulation studies of polybutilcarbosilane dendrimers of various generations in bulk have recently been performed [33,34] to find the microscopic origin of the experimental observations of the liquid-to-solid transition in these systems. An atomistic model developed in [34] has provided a rather good agreement of the simulation results with the available experimental data; in particular, on a weak dependence of the melt density on dendrimer generation, the values of the thermal expansion coefficients and self-diffusion coefficients, as well as the heat capacity at the plateau region, near room temperature. However, the performed structural analysis has not yet allowed us to find any qualitative differences between low- and high-generation dendrimers and to make any conclusions on the physical nature of the experimentally observed phenomena. The further development of this work is envisioned in two directions. First, investigations of the melt dynamics are needed, and they are currently under development. Second, an expansion of the research to other systems with a closer but different structure seems to be promising. The latter can help to find common features in the behavior of the dendrimers owing to their tree-like architecture. Polymethylsilsesquioxane dendrimers are good candidates for this purpose, because they do not have any specific chemical groups in their composition and are characterized by a low energy of interchain interactions as are the carbosilane dendrimers. We have already studied the structural characteristics of two homologues series of trimethylsilyl derivatives of polymethylsilsesquioxane dendrimers [35]. An analysis of the radial mobility of their terminal groups has shown that long trajectories of tens of nanoseconds are required for a proper study of dendrimer intramolecular dynamics.

In this paper, we focus on the comparative study of the dynamics realized in single siloxane and carbosilane dendrimer molecules of the fourth to the seventh generation. There is little research on intramolecular dynamics in dendrimers. One should mention a few studies considering the orientational segmental mobility within the coarse-grained approaches [36,37,38,39] and semi-atomic studies of the local conformational mobility of dendrimer units for carbosilane dendrimers of the fifth generation [40,41]. In this paper, we study two types of siloxane dendrimers, which differ by the length of the spacers, and two types of the carbosilane dendrimer with different functionality of the core atom. The importance of this research is twofold. First, it gives some insight into the effect of the structural parameters of the dendrimers on the local dynamics of their branching atoms. Second, the obtained results would serve as a basis for future investigations of the local dynamics in their melts.

## 2. Materials and Methods

In this section, we describe in detail the structure of the dendrimers under study, the method of simulation, as well as the approaches to the analysis of the intramolecular dendrimer dynamics.

### 2.1. Dendrimer Structures

**Figure 1:**
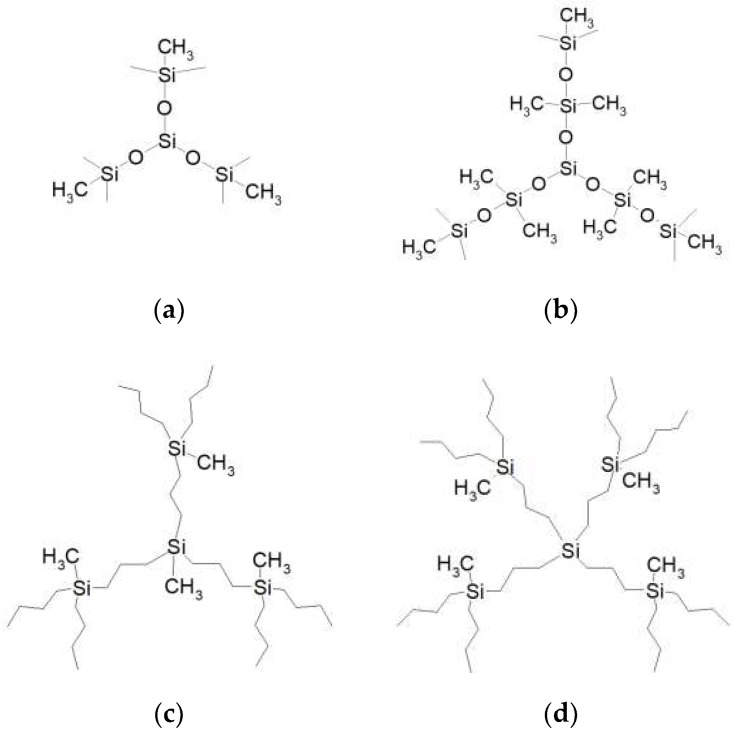
The structure of the first generations of the dendrimers under study. (**a**) siloxane dendrimers with shorter spacers (s-dendrimers), (**b**) siloxane dendrimers with longer spacers (l-dendrimers), (**c**) polybutylcarbosilane dendrimers with three-functional Si core atoms (c3-dendrimers) and (**d**) polybutylcarbosilane dendrimers with tetrafunctional Si core atoms (c4-dendrimers).

We model two families of silicone-containing dendrimers, each of them is represented by two types of dendrimer molecules with different molecular structures. The first family is of siloxane nature, their representatives are shown in Figure 1a,b. Both of them have a trifunctional Si-(CH_3_) core and trifunctional branching Si atoms, while the terminal segments are methyl groups. The difference is in the length of spacers. The siloxane dendrimers of the first type have short -O- spacers (Figure 1a), while the spacers -O-Si(CH_3_)_2_-O- of the second-type of siloxane dendrimer are longer (Figure 1b). For convenience, we denote the siloxane dendrimers with the shorter and longer spacers as s- and l-dendrimers, respectively.

The second family under study consists of similar carbosilane dendrimers with -(CH_2_)_3_- spacers and -(CH_2_)_3_-CH_3_ terminal groups but belonging to the different homologue rows with three- and tetrafunctional Si core atoms. The structure of the first-generation carbosilane dendrimers is schematically shown in Figure 1c,d, respectively. We shall use the abbreviations c3-dendrimers and c4-dendrimers for these two types of carbosilanes.

Thus, the object of our study are four types of dendrimers, which can be divided into three pairs for a reasonable comparison. First, a comparative analysis of two siloxane dendrimers can give us some information on the effect of the spacer length on the dendrimer behavior. Investigations of equilibrium properties of these dendrimers have been performed in [35]. Here, we focus on some differences in their dynamic behavior. Second, the siloxane l-dendrimers and carbosilane c3-dendrimers have almost identical spacer lengths but differ in chemical composition and thus in the type of chemical bonds. One can expect that the different rotational mobility of Si-O and Si-C bonds would contribute to the distinct dynamics of the corresponding dendrimers. Finally, the two carbosilane c3- and c4-dendrimers differ in the core functionality defining the density of their molecular structures and thus the main features of their intramolecular dynamics.

To specify the generation number of a particular dendrimer under discussion, we use the notation Gi, where i is the generation number.

### 2.2. Model

We perform molecular dynamics simulations of dendrimer molecules within the PUMA software package [42,43]. The siloxane dendrimers are modeled by the polymer consistent force field (PCFF) [44], while the AMBER force field [45,46] is used in the simulations of carbosilane dendrimers. The AMBER force field is an appropriate one for simulations of carbon-chain polymers; however, it does not contain potentials for modeling Si-O bonds, which are supported by the PCFF. To make a comparison between these two families of dendrimers feasible, the interaction parameters for the siloxane dendrimers were taken from the PCFF in the same functional forms as in the AMBER force field. We believe that the substantially different type of Si-O bonds in the siloxane dendrimers plays a major role in their dynamics and determines the major differences with the carbosilane dendrimers. Potentials describing bond stretching and bond bending are accounted for in both dendrimer families, and the potential of the dihedral angle rotation around the equilibrium values is additionally used for the carbosilane dendrimers. Non-bonded interactions are expressed via the Lennard-Jones (LJ) potential with the cutoff distance set to 1.05 nm. In addition, electrostatic interactions arising from partial charges on the constitutive atoms are also taken into account. Contributions to the partial charges on the atoms from redistributions of charges via covalent bonds are taken from the PCFF. Coulomb interactions between atoms with partial charges $q_{i}$ and $q_{j}$ at the distance $r_{ij}$ are calculated using the screened Coulomb potential as follows:

(1) $$U_{q}\left(r_{ij}\right)=\frac{q_{i}q_{j}}{r_{ij}}W_{q}\left(r_{ij}\right).$$

The screening function is as follows:

(2) $$W_{q}\left(r\right)=\{\begin{array}{c} {\left(1-\frac{r}{R_{q}}\right)}^{2},r<R_{q}\\ 0,\;r\geq R_{q} \end{array}$$

where *R_q_* is the screening radius. Its value is chosen to be equal to the cutoff distance of the LJ potential.

Tables S1−S5 in the Supplementary Materials provide all the main parameters of the potentials for the siloxane and carbosilane dendrimers. The same values have also been used in [34,35]. Within the accepted model, we effectively simulated isolated dendrimers in a vacuum. They could serve as a reference system for further simulations of the melts of these dendrimers. Moreover, at room temperature, the adopted values of the interaction potentials roughly describe the dendrimer conformations in a poor solvent. At enhanced temperatures, the dendrimer is expected to swell, because the attractive part of the Lennard–Jones potential becomes insignificant; however, it should be mentioned that this swelling could be different from that in an explicit solvent.

The details of the preparation of the initial non-overlapping dendrimer conformations with correct values of the bond length and valence angles are described elsewhere [40]. Standard molecular dynamics (MD) techniques with a collisional thermostat [47,48] were used for the system relaxation. The elementary integration step was 0.002 ps. The results of our previous work [35] clearly demonstrated that the relaxation of the dendrimer macroparameters is reached after 3 ns for all the generation numbers; in particular, all the potential energy contributions, as well as the radius of gyration or the shape factor, are completely equilibrated. Thus, in this study, equilibration of dendrimers from the fourth up to the seventh generation was performed for 6 ns at T = 600 K. Then, long trajectories of up to 100 ns (200 ns for the generation-7 (G7) s-dendrimers) were obtained for all the types of dendrimers. It has been shown in [35] that a complete mixing of the atoms within the dendrimer interior takes place during 30–80 ns depending on the generation number (i.e., the atoms belonging to the same topological layer became indistinguishable from the point of view of the radial and angular types of motion).

On this long trajectory of 100 ns, the autocorrelation functions for the radial and angular displacements of each Si atom were obtained (see below), and the time necessary for an objective analysis of the corresponding autocorrelation functions was estimated. Thus, to study dynamic characteristics and to calculate relaxation time distributions (described in more detail below), 8 independent samples of each dendrimer type and every generation were simulated at 300 K and 600 K, with the time trajectories of 30 ns, after equilibration for 6 ns, in order to collect more complete statistics. Ensemble averaging was performed for the evaluation of relaxation time distributions.

### 2.3. Radial and Angular Mobility

To study the dendrimer intramolecular dynamics, we decompose the movement of each branching Si atom into radial and angular ones. The radial motion is described by the atom displacements from the center and toward the center of the dendrimer along its radius (i.e., by the time evolution of the distance R from a particular Si atom to the core one).

**Figure 2:**
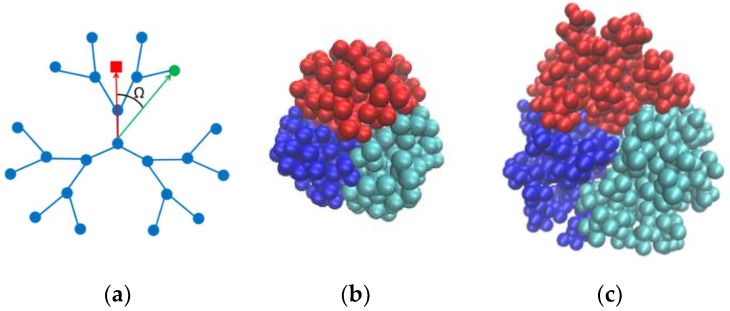
Schematic representation of the angle used to characterize the angular motion of Si branching atoms. The green color indicates the Si atom being studied, and the red square indicates the center of mass of the corresponding dendron (**a**). A balanced molecule of generation-6 (G6) s-dendrimer (**b**) and l-dendrimer (**c**). The different colors in (**b**,**c**) are painted atoms belonging to different dendrons.

The angular motion of Si atoms is described by the dynamics of the angle Ω between two vectors drawn (i) from the central atom to the atom under consideration and (ii) from the central atom to the center of mass of the dendron to which the atom belongs (see Figure 2). This method was chosen to unambiguously exclude the effect of a possible rotation of the dendrimer molecule as a whole. It should be noted that the average value of the angle Ω and its dispersion can give us some information on the degree of dendron mixing within dendrimer molecules and allow a qualitative comparison of dendron interpenetration in dendrimers of various generation numbers and structures. In particular, a decreasing Ω together with its dispersion indicates a weaker overlap of dendrons. The degree of dendron mixing can also be estimated via snapshot visual analysis. Snapshots of G6 s- and l-dendrimer molecules presented in Figure 2b,c demonstrate that the dendrons overlapping in these molecules are small (snapshots of the other dendrimers are shown in Figure S1 of the Supplementary Materials).

**Figure 3:**
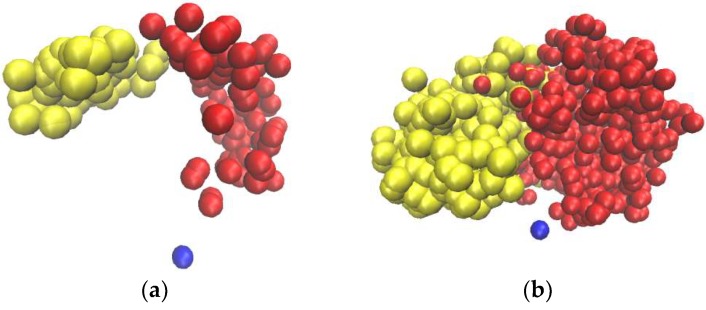
The positions (every 10 ps) of two Si terminal atoms (colored in yellow and red) of the G7 l-dendrimer, during 1 ns (**a**) and 10 ns (**b**). The central atom of the dendrimer is painted blue.

It should be stressed that the averaging time is fundamentally important for the calculation of the radial and angular position averages. This fact is clearly demonstrated in Figure 3, where the snapshots of the positions of the two terminal Si atoms belonging to the same branching layer but different branches of a generation-7 (G7) l-dendrimer are shown for 1-ns and 10-ns trajectories. Different atoms of one branching layer, or one topological distance, have completely different radial and angular mobility on the shorter trajectory. In particular, after 1 ns of the system evolution, the radial displacement of the atoms shown by the yellow is very small in comparison with the one of the red atoms. They have also different angular mobility. Our measurements show that the characteristic times necessary for all the atoms to become indistinguishable from the point of view of these statistics are tens of nanoseconds, which is an order of magnitude higher than the relaxation time of the molecule as a whole (in particular, the relaxation times of the gyration radius of the carbosilane and siloxane dendrimers were calculated in [34,35], respectively). Indeed, one can see in Figure 3b that after 10 ns, the areas covered by the red and yellow atoms in the course of their motion are practically identical. The radial mobility of the terminal groups of siloxane dendrimers has been studied in [35], where it has already been mentioned that there are different populations of terminal groups with enhanced and reduced radial mobility. In this paper, we show that a similar situation takes place with carbosilane dendrimers; furthermore, tens of ns are needed not only for radial but also for angular mixing.

It should be mentioned that the interpretation of the behavior of the dendrimer as a sequence of spherical layers with different dynamics has been described in [36] within a coarse-grained approach.

### 2.4. Autocorrelation Functions and Relaxation Time Distributions

The autocorrelation functions were used to study the characteristic relaxation times of the radial and angular motions of all the Si atoms excluding the core one. The autocorrelation functions were calculated according to the following expression:

A(t) = (<δX(t)∙δX(0)>)/(<δX^2^>) (3)

with

δX(t) = X(t) − <X>(4)

where X is either the radial coordinate R or the value of the angle Ω of the atom being studied.

**Figure 4:**
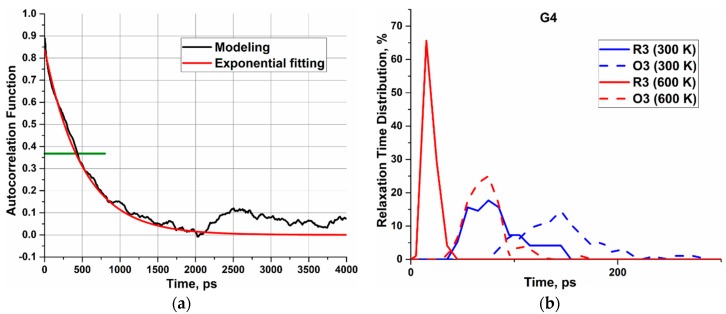
(**a**) The example of the angular autocorrelation function of one of the terminal groups of the generation-6 (G6) s-dendrimer. The green line indicates the value of 1/e. (**b**) The example of the relaxation time distribution for the angular (O) and radial (R) motion of Si atoms belonging to the third layer of the generation-4 (G4) s-dendrimer. The red color corresponds to 600 K, and the blue curves are calculated at 300 K. The solid and dashed curves show distributions of relaxation times for the radial (R) and angular (Ω), respectively.

The autocorrelation functions can be nicely fitted by one exponential function. Thus, the relaxation time is defined as the time at which A(t) reaches the value of 1/e. As an example, an angular autocorrelation function of a terminal group of the G6 s-dendrimer and its exponential fitting are shown in Figure 4a. In this particular case, the characteristic relaxation time is about 490 ps.

Relaxation times of the radial and angular motions of all Si atoms for all types of siloxane and carbosilane dendrimers of the fourth to the seventh generations were calculated from the autocorrelation functions. Relaxation time distributions for Si atoms belonging to a particular branching layer were obtained via averaging over all the atoms of the same branching layer and over eight ensembles of dendrimers. As an example, the corresponding distribution calculated for the third layer of the fourth generation of the s-dendrimer is shown in Figure 4b. Given the large number of histograms, we will further analyze the dynamics of Si atoms using mean values of relaxation times together with the corresponding variances.

Furthermore, we have analyzed the dynamics of the torsional angles of all the dendrimers under study.

## 3. Results and Discussion

### 3.1. Radial and Angular Positions of Si Branching Atoms

Let us discuss first the results on the radial and angular motion of the Si atoms within the dendrimer molecules of various types.

**Figure 5:**
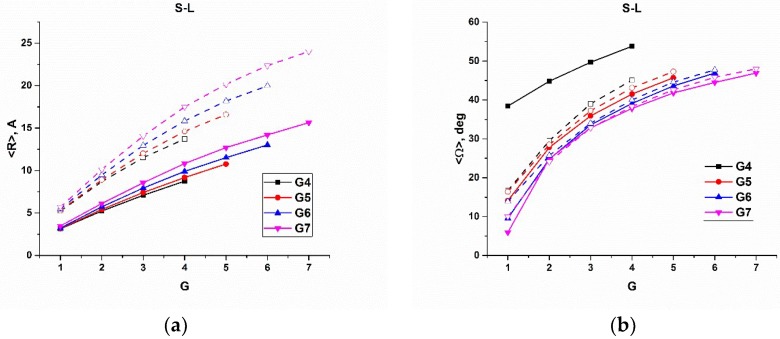

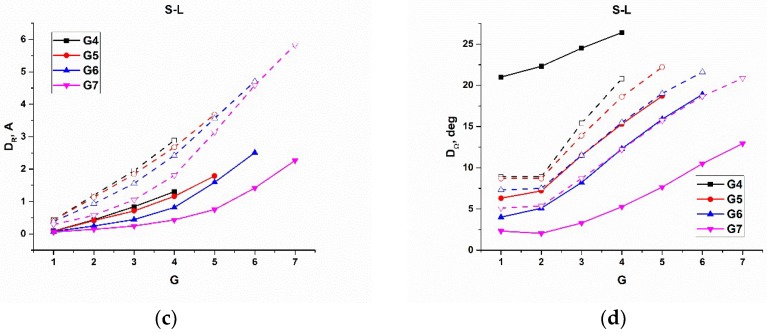
(**a**) The average distance, <R>, of the Si branching atoms to the core atom, (**b**) the average angle, <Ω>, between the Si atom and the center of mass of the dendron (**c**) the square root of the R dispersion, D_R_, and (**d**) the square root of the Ω dispersion, D_Ω_, calculated for s-dendrimers (solid lines) and for l-dendrimers (dashed lines).

**Figure 6:**
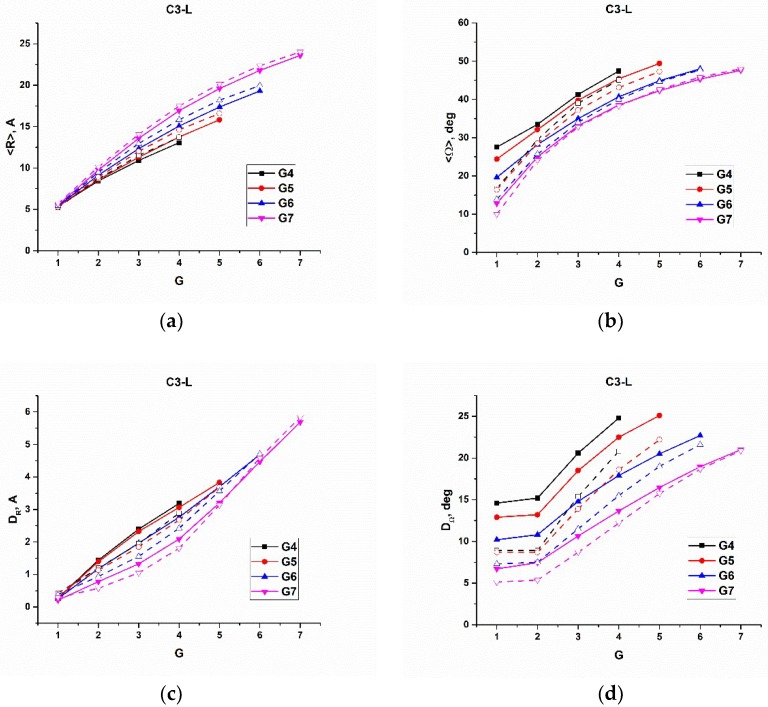
(**a**) The average distance, <R>, of the Si branching atoms to the core atom, (**b**) the average angle, <Ω>, between the Si atom and the center of mass of the dendron (**c**) the square root of the R dispersion, D_R_, and (**d**) the square root of the Ω dispersion, D_Ω_, calculated for c3-dendrimers (solid lines) and for l-dendrimers (dashed lines).

**Figure 7:**
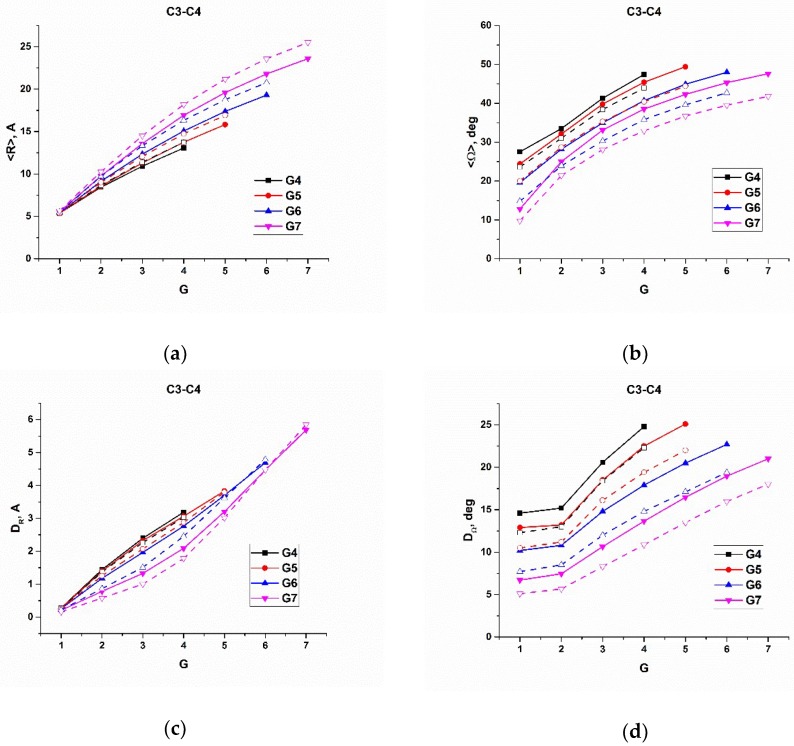
(**a**) The average distance, <R>, of the Si branching atoms to the core atom, (**b**) the average angle, <Ω>, between the Si atom and the center of mass of the dendron (**c**) the square root of the R dispersion, D_R_, and (**d**) the square root of the Ω dispersion, D_Ω_, calculated for c3-dendrimers (solid lines) and for c4-dendrimers (dashed lines).

In Figure 5, Figure 6 and Figure 7, we plotted the average radial distance, <R>, of Si atoms from the dendrimer core, and the average angle, <Ω>, of Si atoms with respect to the center of mass of the corresponding dendrons, as well as their variations, versus the number of the branching layers to which the Si atoms belong, calculated for the all the types of dendrimers of various generations on 100 ns trajectories at 600 K. As has been mentioned above, the time of 100 ns is long enough for the Si atoms of each layer to be almost indistinguishable in their mobility characteristics, and the averaging was performed within each branching layer. For the reasonable comparison, we sorted out the results into three series. In particular, Figure 5 shows the corresponding dependences for the s- and l-dendrimers differing by the spacer length. Figure 6 demonstrates the effect of the chemical nature of the dendrimer molecules through the comparison of l- and c3-dendrimers, while the results for c3- and c4-dendrimers shown in Figure 7 allow us to make some conclusions on the influence of the core atom functionality. The corresponding plots of another indicative characteristic, which is the difference between the maximum and minimum values of the radial distance (and angle), R_max_–R_min_, (and Ω_max_–Ω_min_) are presented in the Supplementary Materials, Figures S2–S4.

**Figure 8:**
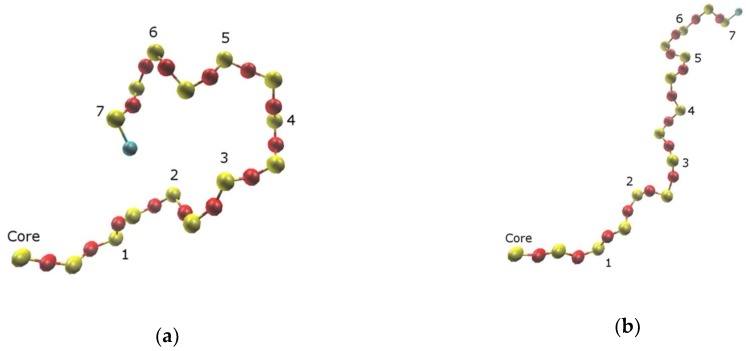
Two instant conformations (**a**,**b**) of one isolated linear chain of the generation-7 (G7) l-dendrimer, from the core Si atom to the terminal group, taken within an interval of 100 ps. The Si branching atoms are numbers from the core.

First of all, one can see in Figure 5, Figure 6 and Figure 7 that the Si atom behavior was qualitatively quite similar for all the types of dendrimers. In particular, with increasing the generation number, the average radial position of Si atoms realized within a given branching layer increased, while the average angle decreased. At the same time, the allowable value corridor (i.e., the difference between the maximum and the minimum values of the radial position), as well as the angle, became narrower. We directly looked at the maximum and minimum values of these parameters, and we concluded that the radial corridor was compressed to the maximum values and the angular one to the minimum values. This means that the dendrons were stretched from the center and squeezed in the solid angle. An enhanced angular mobility of Si atoms of generation-4 (G4) s-dendrimers (Figure 5b,d) was due to the absence of any significant excluded volume restrictions. The space-filling effect was not yet manifested for this dendrimer with the small number of atoms. It should also be noted that the variations of both R and Ω stayed rather small for two internal layers of all generation dendrimers, except for G4 s-dendrimers, and then started to grow rather rapidly. The variation of R increased more rapidly for high generation dendrimers, while the Ω variation growth slowed down with the generation number (i.e., the angular mobility of the outer layers decreased with generation). Visual examination of the movement of a given dendrimer chain from the core atom to one of the terminal groups indeed showed that the Si atoms of a few internal topological layers just slightly fluctuated around their average positions, while the bending of the chain usually occurred starting from the third topological layer (snapshots of bent and stretched conformations of a G7 l-dendrimer chain are shown in Figure 8, while the movie demonstrating its movement is available in the Supplementary Materials, Video S1). This conclusion was also supported by a sharp increase of D_Ω_ at the third layer (G = 3 in Figure 5d), as well as by the distributions of the Si branching points belonging to various layers with respect to the dendrimer center of mass calculated in [35]. The maxima of the distributions for the Si atoms belonging to the first two layers were well separated, while some layer mixing occurred starting from the third layer.

If we look at the positions of the terminal groups in Figure 5, Figure 6 and Figure 7, one can see that their radial distance from the core naturally increased with the generation number and so did the dispersion of R and the value of R_max_–R_min_. This fact corresponds well to the well-known backfolding phenomenon [20]. The angular behavior of the terminal groups with the generation number was quite different; namely, the average value stayed practically constant, but its variation and Ω_max_–Ω_min_ decreased considerably with G. The latter fact was more pronounced for the carbosilane dendrimers.

The tendencies mentioned above were typical for all the dendrimers under study; however, the absolute values of the corresponding parameters depended on the dendrimer structure. In Figure 5, one can see that the increase of the spacer length caused some increase of <R> value for all branching layers but had a minor effect on the average angle except for the G4 dendrimers. On the other hand, the variations of both parameters grew considerably for l-dendrimers (i.e., the longer spacer gave more freedom for Si atoms in both angular and radial motions). It is natural that the largest difference was realized for the outer layers.

The c3- and l-dendrimers were expected to have a comparable length of the spacers but different bond structures; thus, one can see in Figure 6 that indeed, the average radial positions of Si atoms were practically the same in these two types of dendrimers with <R> being a bit smaller for the c3-dendrimers. However, the difference in the average angle and especially in the angle variation was rather big. The inner layers of the carbosilane c3-dendrimers have more angular mobility. Perhaps this difference was due to the presence of two methyl groups on the Si atom in the center of the l-dendrimer spacers, which occupied some additional volume, caused some stretching of the spacers, and slowed down the mobility of the branching atoms.

Comparing c3- and c4-dendrimers (Figure 7), one can conclude that the increase of the core functionality caused some stretching of the branching layers, a decrease the radial fluctuations of the branching Si atoms, as well as a decrease of their angular mobility due to some growth of the molecular structure density.

In Table 1, we summarize the results on the radial and angular parameters obtained for the terminal groups of the sixth generation of each type of dendrimers. One can see that s-dendrimers, with spacers twice as short as those of the other dendrimers, had a smaller <R> value. On the other hand, l-, c3-, and c4-dendrimers had very close radial characteristics; the larger differences were in their angular behavior. The largest value of Ω, as well as its variation, were realized for the c3-dendrimer. For the c4-dendrimer, the angular motion was limited due to the increased amount of dendrons while the slowing in the angular mobility within the l-dendrimers could be caused by some additional restrictions due to the presence of two methyl groups in their spacers.

As for the dendrons themselves and the dynamics of the angle between them, it was determined by the symmetry and steric constraints. Accordingly, with increasing generation, the magnitude of the fluctuations decreases. For example, for all dendrimers with a three-functional core, the values of the angles between the dendrons fluctuated about 120 degrees. The fourth generation had the largest spread, up to 30 degrees, and the values themselves were slightly lower. For the seventh generation, the deviations were very small, only a few degrees. For the c4-dendrimers, there were six angles between the dendrons, and they could be divided into two “big” and four “small” ones. Changes in these angles were clearly correlated with each other. The correlation coefficients were calculated from the time dependences of all the angles presented in the Supplementary Materials, Figure S5. The values of the correlation coefficients for the dendrons with the in-phase and anti-phase movements within the G7 c4 molecules were in the range of 0.41–0.56 and −0.12–−0.7, respectively. As for the deviations, their behavior was identical to the other types of dendrimers, but for “big” angles, the corresponding values were several degrees higher. The characteristic values were 27 degrees for the fourth generation and 8 degrees for the seventh generation. We should also note that the dynamics of these angles do not show so much the movement of a branch as a whole but rather a constant redistribution of mass inside the dendron.

### 3.2. Relaxation Time

**Figure 9:**
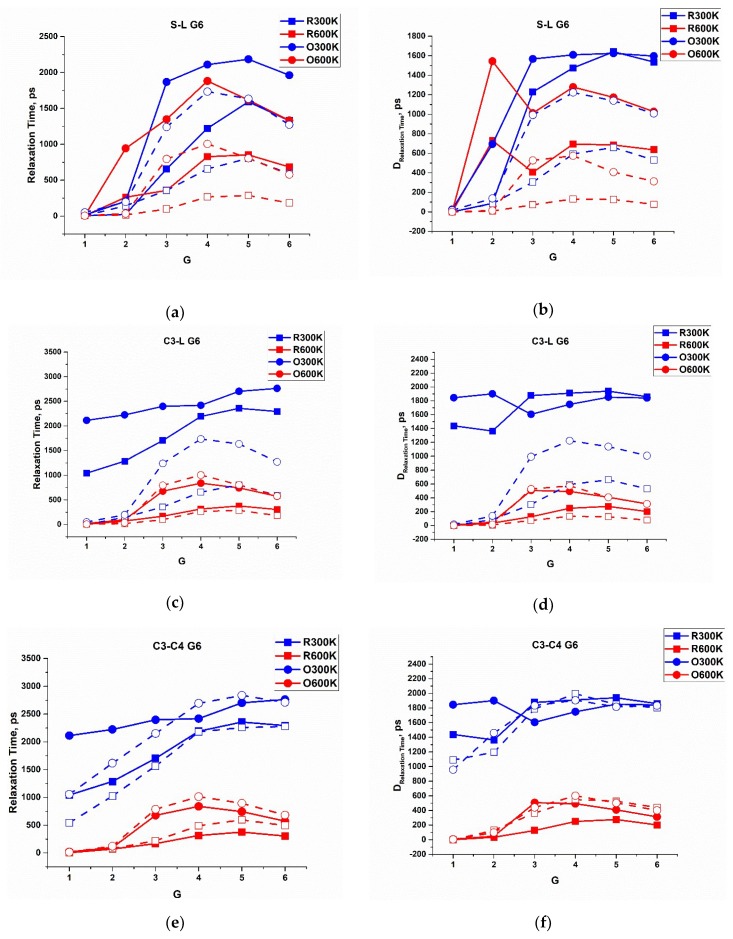
Dependences of the mean relaxation times (left column) and their variations (right column) for the radial (blue) and angular (red) motion of Si branching atoms on the branching layer number for the six generation of s- (solid line) and l-dendrimers (dashed line) (**a**,**b**); l- (solid line) and c3-dendrimers (dashed line) (**c**,**d**); and c3- (solid line) and c4-dendrimers (dashed line) (**e**,**f**), at 300 and 600 K.

As it was mentioned in Section 2.4, it is convenient to perform the analysis of the relaxation time distributions for the radial and angular motion of Si atoms through their mean values, τ, and dispersions, D_τ_. They were calculated for all the types of dendrimers under study. In Figure 9, we plotted the corresponding graphs for the G6 dendrimers in the same comparative way as in the previous section. In particular, we compared the mean relaxation times for the s- and l-dendrimers (Figure 9a,b), for the l- and c3-dendrimers (Figure 9c,d), and for the c3 and c4-dendrimers (Figure 9e,f). The corresponding plots for the dendrimers of all other generation numbers can be found in the Supplementary Materials, Figures S6–S8.

First of all, it is worth noting that the variations of τ were very large, being even comparable with the mean values themselves, indicating a fairly broad nature of the distributions. Second, for all the types of molecules, the relaxation of the angular motion was slower than the radial one. Third, the characteristic relaxation times of all Si atoms increased with an increasing generation number and also from the core layer to the periphery. Nevertheless, the periphery itself (one or more terminal layers) relaxed more rapidly, which was especially noticeable for the siloxane dendrimers. As a result, most of the τ dependences had a maximum located at the intermediate branching layers. This maximum shifted to the terminal layer with an increasing dendrimer generation. Finally, an increase in temperature accelerated relaxation, but for large generations of siloxane dendrimers, this effect was less pronounced or reversed, as in the case of the G7 s-dendrimer. Perhaps, this later fact is due to the very stressed conformations of the G7 s-dendrimer (the conformational analysis has been performed in [35]).

It is worth noting that the characteristic order of the relaxation times was fundamentally different in case of siloxane and carbosilane dendrimers. If we compare the sixth generations, the mean relaxation time of the Si atoms belonging to the first two layers of both the s- and l-dendrimers was very low, while it was larger than 500–1000 ps for the carbosilane molecules. It should be noted that different force fields were used for modeling of siloxane and carbosilane dendrimers; however, owing to the same functional form of the potentials and a proper parametrization, we cannot expect that this fact significantly affected their comparative dynamics. The difference in relaxation times for siloxane and carbosilane dendrimers was due to the different nature of the chemical bonds in these two types of molecules rather than any impact of the force fields used.

### 3.3. Rotational Angle Dynamics

In addition, we performed the analysis of the rotational angle dynamics for all the types of dendrimers. To characterize the conformational mobility of the bonds, we calculated the frequency of the transitions between the trans and gauche isomers. For each torsion angle, the whole angular space was divided into three equal regions near the energy minima. We considered that a transition between the trans and gauche states (between neighboring angle regions) takes place if the bond remains in the new state (new angle region) longer than 0.4 ps.

**Figure 10:**
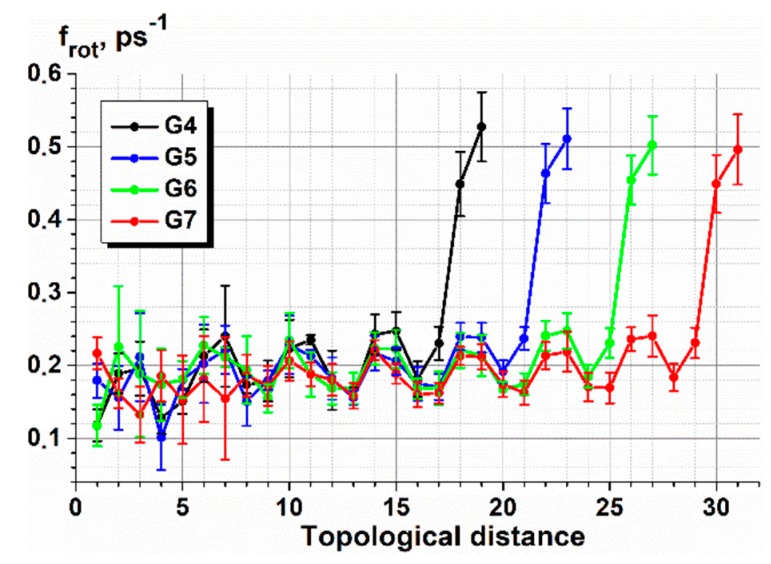
The average frequency of the transitions between the trans and gauche conformations vs. the number bonds (calculated from the core Si atom) for the c3-dendrimers of various generation numbers at 300 K.

Due to the difference of the bond nature in the carbosilane and siloxane dendrimers, the rotational dynamics of bonds is quite different for these two dendrimer families. Let us discuss first the carbosilanes. In Figure 10, we plotted the average frequency of the transitions between the trans and gauche conformations for all the dendrimer bonds of the G4–G7 c3-dendrimers (the corresponding plots for the other dendrimers are shown in Figure S9 of the Supplementary Materials). The averaging was performed among all the bonds corresponding to a given topological distance from the core Si atom. One can see a clearly expressed alternation of the values of this quantity along the topological distance. Two subsequent smaller values corresponded to the C-Si and Si-C consecutive bonds, which were less mobile than the C-C bonds, demonstrating a higher frequency of transitions between the rotational states.

**Figure 11:**
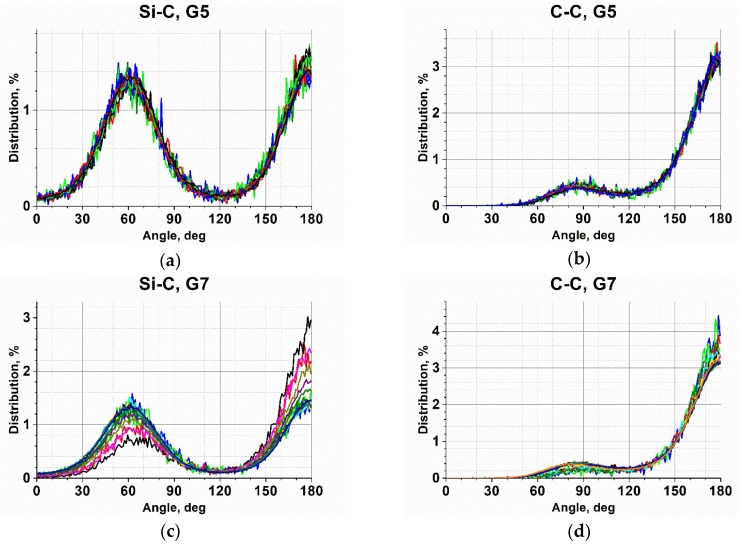
The distribution function of the rotational angle for the Si-C (**a**,**c**) and the C-C bonds (**b**,**d**) for the c3-dendrimers of the fifth (**a**,**b**) and seventh (**c**,**d**) generations at 300 K.

**Figure 12:**
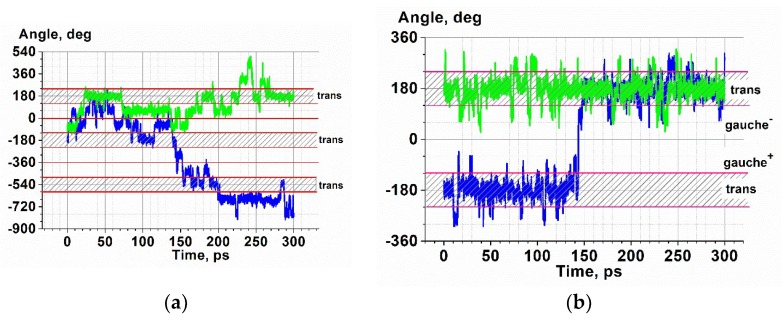
Time evolution of the rotational angles around the Si-C bonds (**a**) and around the C-C bonds (**b**) for sixth layer of the c3-dendrimers of the sixth generation.

This conclusion is confirmed by the angle distributions around the Si-C and C-C bonds (an example of this distribution for the generation-5 (G5) and G7 c3-dendrimers is shown in Figure 11, the corresponding plots for the other generations can be found in the Supplementary Materials, Figures S10 and S11), as well as their time evolution demonstrated in Figure 12 by two arbitrarily chosen angle trajectories. One can see that the probabilities of the trans and gauche conformations of the Si-C bonds of the G5 dendrimer were practically equal (Figure 11a). Indeed, Figure 12a shows that these bonds experienced multiple transitions between the trans and gauche states, but the lifetime in each state was rather long. On the contrary, C-C bonds stayed primarily in the trans conformation but jumped frequently to the gauche conformation; as a result, the angle distribution function had the highest maximum at 180° and a small one at around 90°. The transitions between the trans states through a gauche state, as shown in Figure 12b, were very rare. They were realized only in 4% of trajectories. Thus, one can expect that any bending of the chains took place predominantly via trans-gauche transitions of bonds belonging to the branching Si atoms. Similar behavior was realized in the case of the c4-dendrimers (see the Supplementary Materials, Figure S12).

While for the G4–G6 dendrimers the probabilities of trans and gauche conformation realizations were nearly equal, the relative height of the two maxima of the angle distribution functions was slightly different for the seventh generation of both the c3- (Figure 11c) and c4-dendrimers (Figure S11 in the Supplementary Materials), the rotational angles around the Si-C bonds, which were located after the Si branching point (if we count from the core) in the second and third branching layers, stayed in the trans conformation more often. Also, the С-С bonds of the first and the second topological layers were predominantly in the trans state.

It should also be mentioned that while the mobility of the internal bonds stayed practically constant, the terminal segments appeared to be much more mobile. The frequency of the conformational transitions grew tremendously for two C–C bonds within the methylene terminal segments (Figure 10).

**Figure 13:**
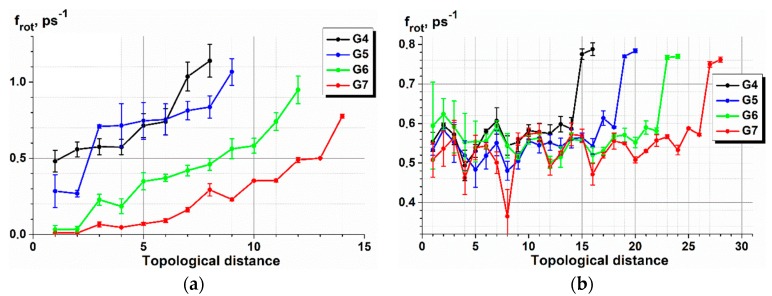
The average frequency of the transitions between the trans and gauche conformations vs. the number bonds (calculated from the core Si atom) for the s-dendrimers (**a**) and l-dendrimers (**b**) of various generation numbers.

Let us now discuss the rotational mobility of the siloxane dendrimers. Their rotational angles were not so well distinguished as those in the carbosilanes; thus, to study their rotational mobility, we calculated the frequency of transitions through 120°. The corresponding plot for the l-dendrimers of various generation numbers is shown in Figure 13b. It resembles the graph in Figure 10 obtained for the c3-dendrimers; however, one can see that the frequency values were much higher for the l-dendrimers. As in the c3-dendrimers, the bonds belonging to the branching Si atoms showed smaller conformational transition frequency than those within the spacers.

The rotational mobility of the bonds of the inner part of the G6–G7 s-dendrimers was considerably suppressed (see Figure 13a), perhaps due to some stretching of the first one or two layers in the sixth and seventh generations. The frequency of the trans-gauche transitions for the bonds of the peripheral layers was as high as that in the l-dendrimers. The highest mobility was realized in the G4 s-dendrimer; it grew fast from the core to the terminal segments.

## 4. Conclusions

In summary, a full-atomic molecular dynamics simulation of the siloxane and carbosilane dendrimers from the fourth up to the seventh generation was carried out to examine the effects of the chemical nature of the bonds, the spacer lengths, and the core functionality on the intramolecular dynamics. The latter was analyzed in terms of the radial and angular displacements of each branching point and the relaxation time spectrum of the radial and the angular motion of the Si branching atoms, as well as the dynamics of the rotational angles belonging to all the bonds of the molecules.

It has been shown that all the dendrimer molecules have some similarities in dynamic behavior. In particular, the dendrons became stretched from the center and decreased in a solid angle with increasing generations. The characteristic relaxation times of the both angular and radial motion increased with increasing generations, with the angular relaxation times being larger than the radial ones. At that, the branching atoms within the intermediate layers relaxed more slowly than those that were close to the core and to the periphery of the dendrimer molecule. Furthermore, the relaxation became faster with enhanced temperatures. It should be stressed that the relaxation time distributions of the majority of the branching layers were very broad with the variation comparable to the mean value, that is, of the order of a few nanoseconds. However, the Si branching atoms of the two first layers, which were the closest to the core, only slightly fluctuated around their average positions, and considerable radial and angular displacements of the Si atoms were realized starting from the third topological layer leading to the backfolding of dendrimer branches.

Comparing the behavior of all the dendrimers under study, one can also find some differences. In general, the dendrimers of the siloxane family relaxed faster than the carbosilane molecules. The s-dendrimers were the most compact among all the dendrimers under study. They had the smallest angular mobility and well-pronounced boundaries between their dendrons. An increase in the spacer length of the siloxane dendrimers gave rise to an enhanced freedom of Si atoms in both the angular and radial motions, which was the best manifested for the Si branching points belonging to one–two terminal layers. The l-dendrimers demonstrated a better mixing of their dendrons in comparison with the s-dendrimers.

The c3-dendrimers were slightly more compact but had a higher angular mobility than did the l-dendrimers in spite of the comparable length of their spacers. This could be caused by the presence of two methyl groups on the Si atoms of the siloxane spacers. However, the relaxation times of both the angular and radial motions were larger for the carbosilanes. An increase of the core functionality made the Si atoms of the c4-dendrimers less mobile; however, the relaxation time of their inner layers became smaller.

There was an unambiguous difference in the rotational mobility of the bonds belonging to the Si branching atoms of the carbosilane dendrimers and those of the spacers. While the frequency of the transitions between the trans and gauche states was higher for the spacer bonds, the bending of the chains took place mainly through rotational isomerism of the Si branching atoms.

These features of the intramolecular dynamics observed in the isolated dendrimer molecules is expected to affect their melt properties. The present study makes a foundation for further investigations of the dynamics of the melt properties of these dendrimers.

## Figures and Tables

**Table 1:** Mean values, the root of the dispersion, and the difference between the maximum and minimum values for the end groups of the sixth generation.

Characteristics	s-Dendrimer	l-Dendrimer	c3-Dendrimer	c4-Dendrimer
<R>, Å	13.0	20.0	19.3	20.8
D_R_, Å	2.5	4.7	4.7	4.8
R_max_−R_min_, Å	12.7	25.6	25.4	25.8
<Ω>, deg.	46.9	47.7	48.0	42.7
D_Ω_, deg.	18.9	21.6	22.7	19.4
Ω_max_−Ω_min_, deg.	109.3	145.3	152.3	130.6

## Supplementary Materials

The following are available online at http://www.mdpi.com/2073-4360/10/8/838/s1, Tables S1–S5: Parameters of the force fields for the atoms; Figure S1: Snapshots of all dendrimers under study; Figures S2–S4: The difference between the maximum and minimum values of the angular and radial positions of the branching Si atoms for all types of dendrimer molecules; Figure S5: Time dependences of the angles between dendron center of mass; Figures S6–S8: Dependences of the mean values and the square root of the dispersion of the relaxation time of each generation layer for all types of dendrimer molecules; Figure S9: The average frequency of the transitions between the trans and gauche conformations vs the bond number for all types of dendrimers; Figures S10–S11: The distribution function of the rotational angle for Si-C and C-C bonds for the c3- and c4-dendrimers; Figure S12: Time evolution of the rotational angles around C-C and Si-C bonds for c3- and c4-dendrimers; and Video S1: Time evolution of the conformation of one of the linear chains from the core of the dendrimer to one of the end groups.
