# Supplementary material for: A Comparative Study of Intramolecular Mobility of Single Siloxane and Carbosilane Dendrimers via Molecular Dynamics Simulations
Source: Polymers (Basel). 2018 Jul 30;10(8):838. doi: 10.3390/polym10080838 (PMC6403907; doi:10.3390/polym10080838)
Supplement: Supplementary file 1 [file polymers-10-00838-s001.zip › Tables_S1_S5_Figures_S1_S12.docx]

Supplementary Materials

A Comparative Study of Intramolecular Mobility of Single Siloxane and Carbosilane Dendrimers via Molecular Dynamics Simulations

Andrey O. Kurbatov ^1,2^, Nikolay K. Balabaev ^3^, Mikhail A. Mazo ^4^ and Elena Yu. Kramarenko ^1,2,^*

1. **Parameters of the force field for the atoms**

**Table S1.** Bond potential ${U_{bond}=\varepsilon}_{b}{(l-l_{0})}^{2}$

| Bond type | ε_b_, kcal∙mol^-1^A^-2^ | $l_{0}$, A |
| --- | --- | --- |
| Si-CH_3_/Si-CH_2_ | 238.0 | 1.809 |
| O-Si | 392.8 | 1.6650 |
| CH_2_-CH_2_/CH_2_-CH_3_ | 322.761 | 1.526 |

**Table S2.** Valence angle potential $U_{angle}{=\varepsilon}_{angle}{(\theta-\theta_{0})}^{2}$

| Atom type | ε_angle_, kcal∙mol^-1^grad^-2^ | $\theta_{0}$, grad |
| --- | --- | --- |
| Si-O-Si | 31.1 | 149.8 |
| X-Si-X | 44.4 | 113.5 |
| X-CH_2_-X | 60.0 | 109.5 |

**Table S3.** Torsion angle potential (for carbosilanes) $U_{tors}{=\varepsilon}_{tors}(1+cos3\varphi)$

| Atom type | ε_tors_, kcal∙mol^-1^ |
| --- | --- |
| X-Si-CH_2_-X | 0.333 |
| X-CH_2_-CH_2_-X | 1.422 |

**Table S4.** Lennard-Jones potential for siloxane dendrimers: $U_{ij}{=\varepsilon}_{ij}\left[ {2\left( \frac{R_{min,ij}}{r_{ij}} \right)}^{9}-{3\left( \frac{R_{min,ij}}{r_{ij}} \right)}^{6} \right]$

$\varepsilon_{ij}={2(\varepsilon_{i}\varepsilon_{j})}^{\frac{1}{2}}\frac{r_{i}^{3}r_{j}^{3}}{r_{i}^{6}{+r}_{j}^{6}}$, $R_{min,ij}={(\frac{R_{min,i}^{6}+R_{min,j}^{6}}{2})}^{\frac{1}{6}}$

| Atom type | ε_i_, kcal∙mol^-1^ | R_min_, A |
| --- | --- | --- |
| CH_3_ | 0.054 | 4.010 |
| Si | 0.070 | 4.284 |
| O | 0.240 | 3.350 |

Lennard-Jones potential for carbosilane dendrimers: $U_{ij}{=\varepsilon}_{ij}\left[ \left( \frac{R_{min,ij}}{r_{ij}} \right)^{12}-{2\left( \frac{R_{min,ij}}{r_{ij}} \right)}^{6} \right]$

$\varepsilon_{ij}={(\varepsilon_{i}\varepsilon_{j})}^{\frac{1}{2}}$, $R_{min,ij}=0.5(R_{min,i}+R_{min,j})$

| Atom type | ε_i_, kcal∙mol^-1^ | R_min_, A |
| --- | --- | --- |
| CH_2_ | 0.1094 | 4.116 |
| CH_3_ | 0.1490 | 4.116 |
| Si | 0.1900 | 4.450 |

**Table S5.** Atomic masses and partial charges of the atoms

Siloxane

| Atom type | m, am u | q, e |
| --- | --- | --- |
| CH_3_-**Si**-3O | 28 | 0.760 |
| 2CH_3_-**Si**-2O | 28 | 0.640 |
| 3CH_3_-**Si**-O | 28 | 0.520 |
| **O** | 16 | -0.440 |
| **CH3** | 15 | -0.100 |

Carbosilane

| Atom type | m, am u | q, e |
| --- | --- | --- |
| CH_3_-**Si**-3CH_2_ | 28 | 0.540 |
| Si-**CH_3_** | 15 | -0.135 |
| CH_2_-**CH_2_**-CH_2_ /CH_2_-**CH_2_**-CH_3_ | 14 | 0.000 |
| CH_2_-**CH_3_** | 15 | 0.000 |

**
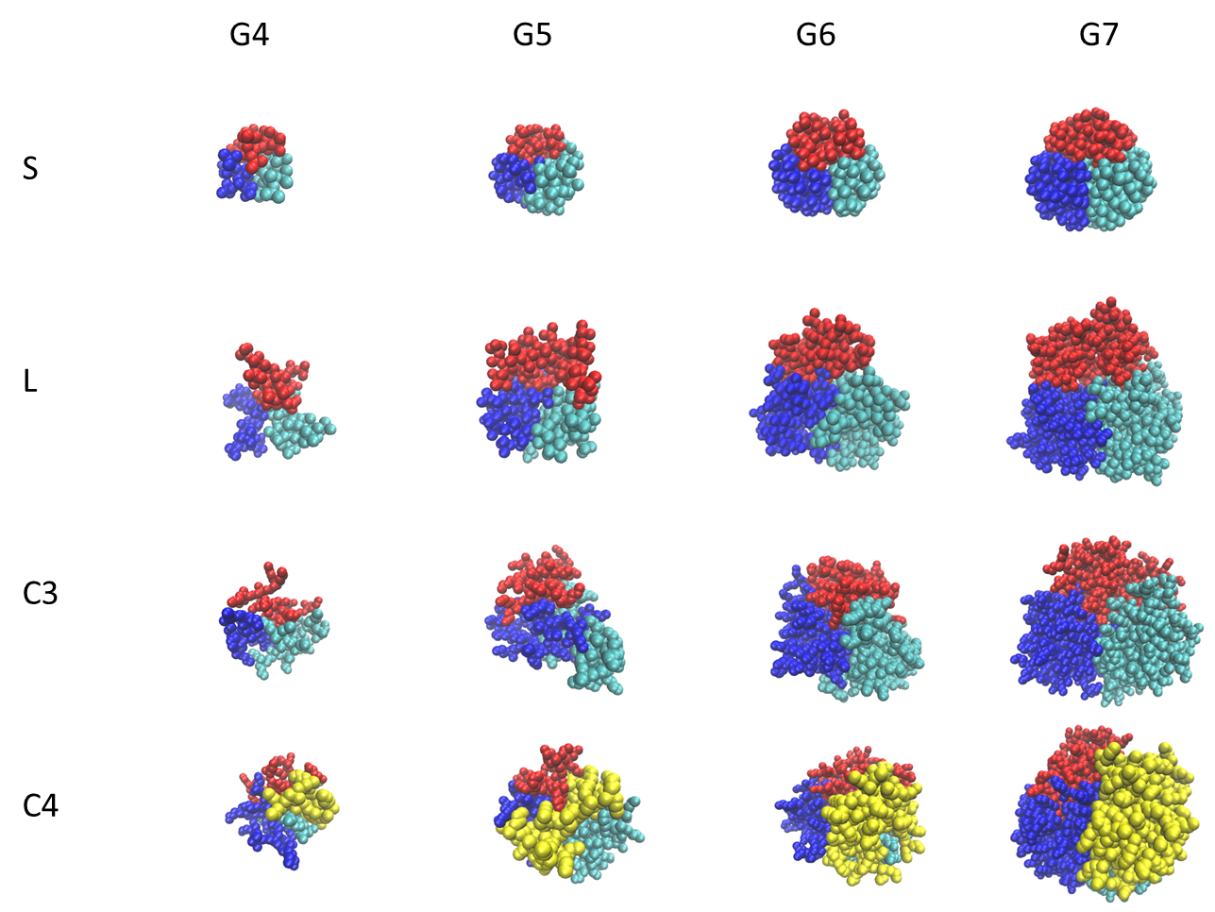
**

Figure S1. Snapshots of all dendrimers under study

1. **Maximum and minimum values of the radial and angular positions of Si branching atoms of various topological layers**

(a) (b)







Figure S2. The difference between the maximum and minimum values (a) of the distance to the center and (b) of the angle between the atom and the center of mass of the dendron for s-dendrimers (solid line) and l-dendrimers (dashed line).

(a) (b)







Figure S3. The difference between the maximum and minimum values (a) of the distance to the center and (b) of the angle between the atom and the center of mass of the dendron for c3-dendrimers (solid line) and l-dendrimers (dashed line).

(a) (b)







Figure S4. The difference between the maximum and minimum values (a) of the distance to the center and (b) of the angle between the atom and the center of mass of the dendron for c3-dendrimers (solid line) and c4-dendrimers (dashed line).

1. **Time dependences of the angles between dendrons**

**

**



**



**

Figure S5. Time dependences of the angles between centers of mass of various dendrons of G4-G7 c4-dendrimers.

1. **Relaxation Time**

(a) (b)







(c) (d)







(e) (f)







(g) (h)







Figure S6. Dependences of the mean values and the square root of the dispersion of the relaxation times of each generation layer for (a, b) G4, (c, d) G5, (e, f) G6 and (g, h) G7 for s-dendrimers (solid line) and l-dendrimers (dashed line), for radial and angular motion for two temperatures.

(a) (b)







(c) (d)







(e) (f)







(g) (h)







Figure S7. Dependences of the mean values and the square root of the dispersion of the relaxation times of each generation layer for (a, b) G4, (c, d) G5, (e, f) G6 and (g, h) G7 for c3-dendrimers (solid line) and l-dendrimers (dashed line), for radial and angular motion for two temperatures.

(a) (b)







(c) (d)







(e) (f)







(g) (h)







Figure S8. Dependences of the mean values and the square root of the dispersion of the relaxation times of each generation layer for (a, b) G4, (c, d) G5, (e, f) G6 and (g, h) G7 for c3-dendrimers (solid line) and c4-dendrimers (dashed line), for radial and angular motion for two temperatures.

1. **Rotation Dynamics**

(a) (b)







(c) (d)







Figure S9. The average frequency of the transitions between the trans and gauche conformations vs the bond number (calculated from the core Si atom) for (a) s-dendrimers, (b) l-dendrimers, (c) c3-dendrimers and (d) c4-dendrimers.

(a) (b)







(c) (d)







(e) (f)







(g) (h)







Figure S10. The distribution function of the rotational angle for Si-C (a, c, e, g) and C-C bonds (b, d, f, h) for the c3-dendrimers. Different colors correspond to different bonds.

(a) (b)







(c) (d)







(e) (f)







(g) (h)







Figure S11. The distribution function of the rotational angle for Si-C (a, c, e, g) and C-C bonds (b, d, f, h) for the c4-dendrimers. Different colors correspond to different bonds.

(a) (b)







(c) (d)







(e) (f)







Figure S12. Time evolution of the rotational angles around C-C bonds for c3-dendrimers (**a**,**b**) and c4-dendrimers (**c**,**d**), and around Si-C bonds for c3-dendrimers (**e**) and c4-dendrimers (**f**) for the 6^th^ layer of the 6^th^ generation.
